# Rapid risk assessment during the early weeks of the 2015‐2016 influenza season in Ukraine

**DOI:** 10.1111/irv.12526

**Published:** 2018-01-15

**Authors:** Sophie Newitt, Alla Mironenko, Olha Holubka, Oleksandr Zaika, Olga Gubar, Katri Jalava, Caroline Brown, Iryna Demchyshyna, Tetiana Dykhanovska

**Affiliations:** ^1^ Public Health England Nottingham UK; ^2^ The L.V. Gromashevsky Institute of Epidemiology & Infectious Diseases National Academy of Medical Science of Ukraine Kyiv Ukraine; ^3^ State Sanitary and Epidemiological Service of Ukraine Kyiv Ukraine; ^4^ Ministry of Health of Ukraine Kyiv Ukraine; ^5^ University of Helsinki Helsinki Finland; ^6^ World Health Organization Regional Office for Europe Copenhagen Denmark; ^7^ Ukrainian Centre for Disease Control and Monitoring of the Ministry of Health of Ukraine Kyiv Ukraine

**Keywords:** influenza A(H1N1)pdm09, risk assessment, seasonal influenza, Ukraine

## Abstract

**Background:**

Several eastern European countries reported a severe influenza season to the World Health Organization (WHO) during late 2015. A country‐specific rapid risk assessment for Ukraine was conducted to assess the season's severity and inform public health action.

**Methods:**

The exposure and hazard were assessed using acute respiratory infection (ARI), severe acute respiratory infection (SARI), laboratory surveillance, virological and vaccine data from weeks 40/2015 to 7/2016 with comparison to 4 previous seasons to describe the influenza season start (5‐week consecutive increase in ARI or SARI), predominant virus types, geographical spread and affected age groups.

**Results:**

The exposure was characterised by an earlier and steeper increase in SARI (week 1/2016) and ARI (week 2/2016) compared to the previous 4 seasons. Transmission was across Ukraine with an increase in ARI and SARI cases aged 30‐64 years compared to 2014/15. Laboratory‐confirmed deaths increased from 11 in 2014/2015 to 342 in 2015/2016; the majority were 30‐64 years old and unvaccinated; and 63.5% had underlying conditions. Total population vaccination coverage was 0.3%. The hazard assessment found influenza virus A(H1N1)pdm09 accounted for >95% of viruses detected. Ukrainian virus strains (n = 62) were antigenically similar to vaccine strains and susceptible to neuraminidase inhibitors.

**Conclusions:**

The first weeks of the 2015/16 influenza season were more severe than previous seasons, with an earlier and steeper increase in severe cases and deaths, particularly in younger adults. Influenza A(H1N1)pdm09 was the predominant strain and was closely related to the seasonal vaccine strain with no evidence of resistance to antiviral drugs.

## 1 INTRODUCTION

Several eastern European countries alerted the World Health Organization (WHO) Regional Office for Europe in late 2015 about a potentially severe influenza season. In response, a rapid risk assessment for the whole WHO European Region was conducted and published in February 2016.^1^, ^2^

There was a high level of concern and media interest regarding influenza in Ukraine, including speculation that the 2009 pandemic influenza virus A(H1N1)pdm09 had become more virulent. Ukraine is a lower‐middle income country in eastern Europe with an estimated population of 45 million.^3^ There has been conflict in eastern Ukraine since 2014, with an estimated 1.5 million internally displaced people within Ukraine.

As part of a WHO Global Outbreak Alert and Response Network (GOARN) response requested by the Ministry of Health of Ukraine, a country‐specific risk assessment for Ukraine was conducted to ascertain the severity of the influenza season, describe the first affected regions and assess the potential impact of the season in order to inform public health action and risk communication for the current and future seasons.

## 2 METHODS

This rapid risk assessment was based on the WHO guidelines on acute public health events.^4^ It was conducted in line with the methodology used for the risk assessment of the 2015/16 influenza season in the WHO European Region.^1^, ^2^

An exposure assessment reviewed acute respiratory infection (ARI), severe acute respiratory infections (SARI) and laboratory surveillance data between weeks 40/2015 and 7/2016 to describe influenza activity compared with 4 previous influenza seasons (2011/12, 2012/13, 2013/14 and 2014/15). We described the initiation (defined as a 5‐week consecutive increase in ARI or SARI^2^), geographical spread, affected age groups, intensity of the influenza season and the predominant circulating virus types. Vaccine coverage was estimated to assess the potential impact of the season. Virological data was reviewed to assess the hazard, including possible evidence of antigenic drift compared to the vaccine strain or acquisition of genetic mutations or antiviral resistance.

### 2.1 Data sources

The Ukrainian Centre for Disease Control and Monitoring of the Ministry of Health of Ukraine (UCDC) has conducted universal acute respiratory infection (ARI) surveillance since 1986.^5^ Information is provided from 16 730 medical institutions covering the whole Ukrainian population, excluding the eastern parts of Ukraine beyond the contact line of the conflict. The standard WHO case definition for ARI was used (Appendix), and laboratory samples were taken on clinical relevance. The number of ARI cases, hospitalisations and deaths by age group was collected each week by 25 regional offices of the State Sanitary and Epidemiological Service of Ukraine. Aggregate figures were reported to the UCDC and submitted weekly to the European Surveillance System (TESSy)^6^ for publication in the joint European Centre for Disease Prevention and Control and WHO Regional Office for Europe bulletin Flu News Europe.^7^ The number of influenza virus detections by (sub)type and population denominators were also reported each week.

Sentinel surveillance for severe acute respiratory infections (SARI) has been conducted by The L.V. Gromashevsky Institute of Epidemiology & Infectious Diseases National Academy of Medical Science of Ukraine in accordance with WHO Regional Office for Europe sentinel guidance for influenza surveillance^8^ since 2007. The system includes 10 sentinel hospitals, located in 4 cities (Kyiv, Dnipropetrovsk, Odessa and Khmelnytskyi)^9^ covering a population of 2.9 million and using the pre‐2011 WHO Regional Office for Europe case definition for SARI (Appendix). Each sentinel site submits six weekly samples for laboratory testing during weeks 40‐20 accompanied with completed case forms. Additional samples were submitted if required. The total number of SARI cases by age group is submitted to TESSy on a weekly basis with the number of samples tested, number of influenza virus detections by (sub)type and population denominators.

In addition to data provided to TESSy, both institutions provided additional subnational data and enhanced case data sets for reported deaths and influenza‐positive SARI cases for 2015/16 and 2014/15 to the WHO Country Office for the risk assessment. Vaccination coverage was also provided by the UCDC, which was collected through physicians and reported to the 25 regional offices each week. Detailed information on virus characteristics for Ukraine was supplied by the WHO Collaborating Centre for Influenza Reference and Research, Francis Crick Institute, London, United Kingdom and WHO Collaborating Centre for the Surveillance, Epidemiology and Control of Influenza, Centers for Disease Control and Prevention, Atlanta, United States of America.

### 2.2 Data analysis

Comparisons were made between the 2015/16 season up to week 7/2016 and the 2014/15 season weeks 40/2014 to 20/2015, unless otherwise stated. Incidence was calculated per 100 000 population using denominators provided by the institutions to TESSy. Proportions for age groups per total cases were calculated with 95% confidence intervals. The proportion of laboratory influenza‐positive samples per total submitted samples each week and the season were calculated. Comparisons of characteristics of cases between seasons were calculated with chi‐squared or Fisher's exact test (if values were fewer than 5),^10^ with 1‐ or 2‐sided tests as appropriate. Analysis was carried out using microsoft excel (2010), STATA release 12^11^ and r (version 3.2.4).^12^

## 3 RESULTS

### 3.1 Exposure assessment

#### 3.1.1 Universal ARI surveillance

**Figure 1:**
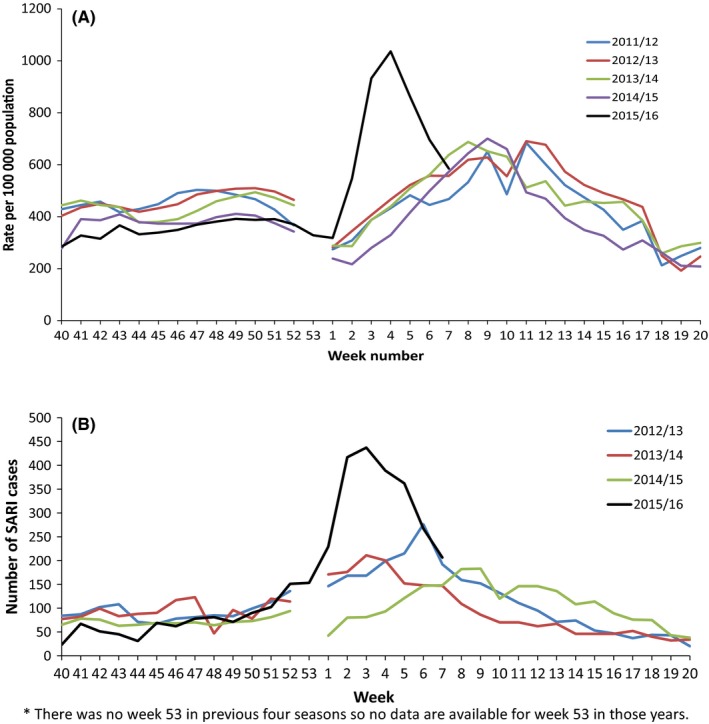
A, Incidence of ARI per 100 000 population for Ukraine, 2015/16 compared with 4 previous seasons*. B, Number of SARI cases reported in Ukraine 2015/16, compared with 3 previous seasons*

Cases of ARI increased from week 2/2016 onwards, earlier than the previous 4 seasons (Figure 1A). Furthermore, the increase in incidence of ARI was much steeper, peaking in week 4 compared to weeks 8‐11 in the previous seasons. The incidence was 1036.6 per 100 000 population during the peak week 4/2016 compared to a peak week of 700.5 cases per 100 000 in week 9/2015. Up to week 7/2016, 4 035 623 ARI cases had been reported, accounting for approximately 9% of the Ukrainian population.

**Figure 2:**
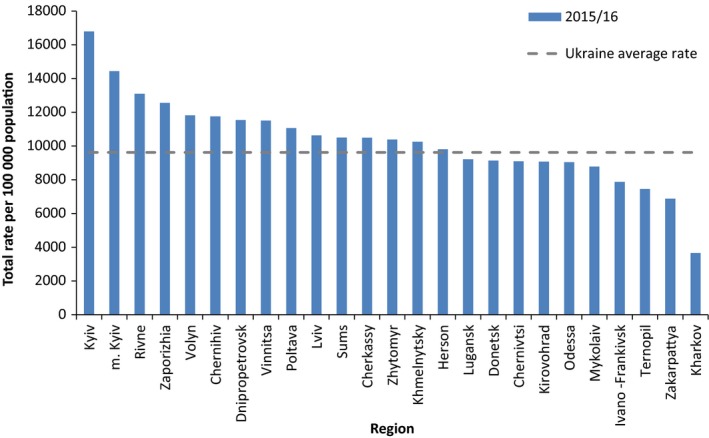
Total incidence of ARI per 100 000 population for week 40/2015 – week 07/2016 for Ukraine, by region

Total ARI rates up to week 7/2016 showed that Kyiv and the surrounding region of Kyiv had the highest incidence (Figure 2). The region of Kharkov (north‐east) had the lowest incidence followed by Zakarpattya and Ternopil (west). The incidence in all regions was higher than the same period for 2014/15 (data not shown).

The Vinnytsia region (south‐west) started to show an increase in ARI from week 51/2015, followed by the Donetsk (east), Odessa (south), Sums (north‐east) and Rivne (north‐west) regions in week 01/2016. The number of ARI cases peaked in most regions in week 3 or 4/2016, with the exceptions of Mykolaiv (south), Zakarpattya (west) and Ivano‐Frankivsk (west), which peaked in week 5.

**Figure 3:**
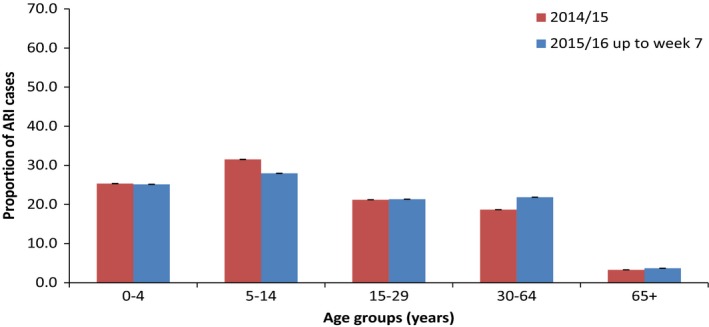
Proportion of ARI cases reported by age group (years) with 95% confidence intervals, in Ukraine 2015/16 and 2014/15

The majority of ARI cases were in those aged <65 years old (96.3%) in 2015/16, similar to 96.7% in 2014/15 (Figure 3). The highest proportion of cases was in the age group of 5‐14 years (28%) for 2015/16 and 2014/15 (31.5%). There was a significant difference between the overall age distribution of cases in 2014/15 and 2015/16 (χ^2^ = 23 000, *P* < .001) with a significant increase in the proportion of cases in the age group of 30‐64 years from 18.7% (n = 1 009 402/5 407 816) in 2014/15 to 21.9% (n = 882 291/4 035 616) in 2015/16 (χ^2^ = 15 000, *P* < .001).

However, the incidence of ARI was highest in the age group of 0‐4 years, which at week 4/2016 was 3183 per 100 000 population. The incidence increased across all age groups from week 2 and all peaked at week 4/2016.

From universal ARI surveillance systems, a total of 5967 samples were submitted for testing up to week 7/2016. Of 1857 influenza‐positive samples, 99.1% were influenza A and 0.86% influenza B. Of the influenza A viruses subtyped (n = 1389), 97.3% were A(H1) and 2.7% A(H3).

For the previous season, 742/3760 samples tested were positive for influenza, 34.0% influenza A and 66.0% influenza B. Of the influenza A viruses subtyped, 48.4% were influenza A(H1) and 51.6% A(H3).

#### 3.1.2 Sentinel SARI surveillance

For 2015/16, SARI cases started to increase from week 49/2015, with a steep increase from week 1/2016 (Figure 1B). Up to week 7/2016, 3381 SARI cases had been reported compared to 1649 cases up to the same point in 2014/15. This was more than the total number of cases reported in the entire 2014/2015 season (n = 3105). SARI cases peaked in week 3/2016, when 437 SARI cases were reported compared to 183 cases at the peak in week 9/2015.

**Figure 4:**
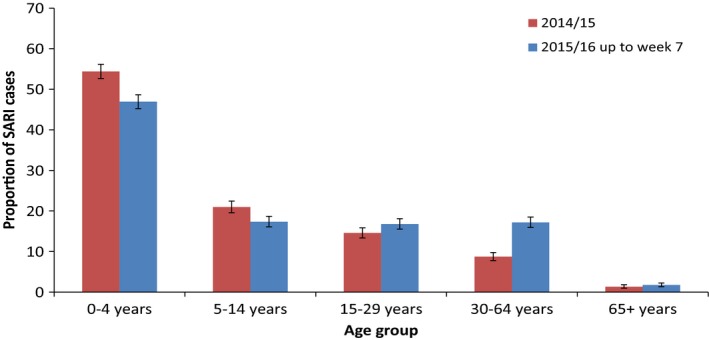
Proportion of SARI cases reported by age group (years) with 95% confidence intervals, in Ukraine 2015/16 and 2014/15

For 2015/16, the majority of the 3381 SARI cases were under 65 years of age (98.3%) (Figure 4). There was a significant difference in the overall age distribution of cases in 2014/15 and 2015/16 (χ^2^ = 123.6, *P* < .001). The proportion of SARI cases that were aged between 30 and 64 years increased significantly from 8.7% (n = 271/3105) in 2014/15 to 17.2% (n = 581/3381) in 2015/16 season **(**χ^2^ = 101.4, *P* < .001).

The area of Kyiv (north) had the highest number of SARI cases in 2015/16. An increase in cases was first seen in Odessa (south) from week 50/2015 peaking in week 1/2016. SARI activity in Kyiv and Khmelnytskyi (west) subsequently peaked in week 3.

**Figure 5:**
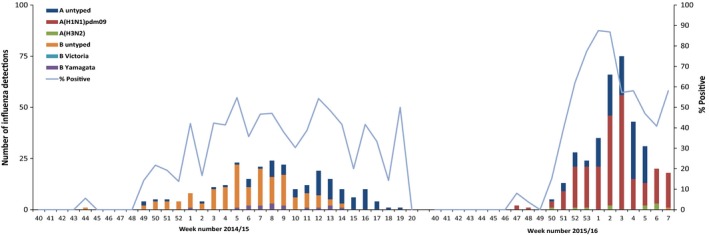
Virus detections among SARI cases by influenza (sub)type Ukraine, weeks 40/2014 to 20/2015 and weeks 40/2015 to 7/2016

Up to week 7/2016, 789 SARI samples had been tested and 361 (45.8%) were positive for influenza. The positivity rate ranged from 3.7% (n = 1/27) in week 48/2015 up to 87.5% (n = 35/40) in week 1/2016 (Figure 5). Of the 361 influenza‐positive samples, 99.7% were influenza A. Of those subtyped (n = 246), 95.9% were influenza A(H1N1)pdm09 and 4.1% influenza A(H3N2). One sample was positive for influenza B in week 7/2016.

In comparison, for 2014/15 838 samples were taken between weeks 40/2014 and 20/2015 and 247 (29.5%) were positive for influenza. The positivity was highest in week 5/2015, at 54.8% (n = 23/42). Of those that were positive, only 34.4% were influenza A (all untyped) and 65.6% were influenza B.

#### 3.1.3 Characteristics of laboratory influenza‐positive SARI cases from sentinel surveillance

Case information was received for 863 sampled SARI cases, of which 381 (44.1%) were positive for influenza up to week 7/2016. For the total 2014/15 season, case information was received for 897 cases, of which 232 (25.9%) were positive for influenza.

During 2015/16 season, 54.6% laboratory influenza‐positive SARI cases were male and the highest proportion of cases was among those aged 30‐64 years (31.2%) (Table 1). However, there was no significant difference in the overall age distribution of influenza‐positive cases compared to 2014/15 (χ^2^ = 4.41, *P*‐value = .354). The highest proportion of positive cases was in Kyiv City (39.6%) compared to the 3 other cities.

**Table 1:** Characteristics of SARI influenza‐positive cases for 2014/15 compared to 2015/16

	SARI patients ‐influenza positive by season n (%)	
	2014/15 (week 40‐20)	2015/16 (up to week 7)
Total SARI patients (n)	232	381
Median time (in days) between onset of symptoms and hospitalization and interquartile range	1.5 (1‐3)	1 (1‐2)
Age (y)		
0‐4 y	45 (19.4%)	81 (21.3%)
5‐14 y	51 (22.0%)	73 (19.2%)
15‐29 y	65 (28.0%)	98 (25.7%)
30‐64 y	60 (25.92%)	119 (31.2%)
65+ y	11 (4.7%)	10 (2.6%)
Median age (interquartile range)	18 (6‐34)	22 (5‐35)
Sex		
Males (% male)	125 (53.9%)	208 (54.6%)
City of hospital/clinic		
Kyiv	79 (34.1%)	151 (39.6%)
Dnipropetrovsk	37 (15.9%)	70 (18.4%)
Odessa	45 (19.4%)	63 (16.5%)
Khmelnytskyi	71 (30.6%)	97 (25.5%)
Any underlying medical condition	33 (14.2%)	69 (18.1%)
Heart disease	10 (4.3%)	9 (2.4%)
Asthma	0 (0%)	3 (0.8%)
Chronic lung disease	1 (0.4%)	5 (1.3%)
Chronic liver disease	0 (0%)	1 (0.3%)
Pregnancy	6 (2.6%)	15 (3.9%)
Chronic kidney disease	2 (0.9%)	1 (0.3%)
Stage II‐III obesity	22 (9.5%)	42 (11.0%)
Diabetes	0 (0%)	6 (1.6%)
Outcome		
Deaths	0 (0%)	9 (2.4%)
Provisional diagnosis		
ARI	121 (52.2%)	61 (16.0%)
Influenza	104 (44.8%)	298 (78.2%)
Pneumonia	5 (2.2%)	22 (5.8%)
Acute bronchitis	1 (0.4%)	0 (0%)
Other	1 (0.4%)	0 (0%)
Influenza vaccination received for season	0 (0%)	0 (0%)
Antiviral treatment	90 (38.8%)	155 (40.7%)

In 2015/16, 18.1% of influenza‐positive cases were recorded as having an underlying medical condition, which was a non‐significant increase from 14.2% in 2014/15 (χ^2^ = 1.569, *P*‐value = .210). The most common underlying medical conditions recorded were stage II‐III obesity (11.0%) and pregnancy (3.9%). None of the influenza‐positive cases had received the seasonal influenza vaccine and 40.7% had been treated with antivirals during their illness (influenza or non‐influenza‐specific antivirals).

In 2015/16, 9 influenza‐positive deaths were recorded (2.4% of all positive SARI cases) compared to no deaths in 2014/15. Two of these deaths were confirmed as influenza positive post‐mortem. Six deaths were aged 30‐64 years, two 0‐4 years and one 15‐29 years. An underlying medical condition was recorded for 44.4% of deaths (n = 4), and the preliminary diagnosis was pneumonia for 66.7% (n = 6). The first death was recorded in Kyiv in week 47/2015, followed by 2 deaths in Odessa in weeks 50 and 52/2015. Four of the 9 influenza‐positive deaths (44.4%) received antivirals during their illness.

#### 3.1.4 Mortality (universal reporting)

Up to week 7/2016, 342 influenza‐positive deaths had been reported to the UCDC from the total Ukraine population. The first death was reported in week 48/2015 from the Kyiv region. The number of influenza‐positive deaths then increased to a peak of 77 in week 4/2016. This was an increase from 11 influenza‐positive deaths reported for the whole 2014/15 season, which occurred between week 6 and week 19/2015.

The majority of influenza‐positive deaths were in the age group of 30‐64 years (82.8%), with most aged between 40 and 59 years. Only 2 deaths were in children <5 years old. The highest proportions of deaths were recorded in Odessa (12.3%), Kyiv region (11.1%) and Donetsk (9.1%).

In 2015/16, 99.4% of deaths were positive for influenza A viruses and of those subtyped (n = 278, 81.8%), 99.3% were influenza A(H1N1)pdm09 and 0.72% A(H3N2). Two deaths were positive for influenza virus B. In 2014/15, 63.6% of the deaths were influenza A positive and 36.4% were influenza B. Of those subtyped (4 of 7) in 2014/15, all were virus A(H1N1)pdm09.

Of the 342 influenza‐positive cases that died in 2015/16, 320 cases were admitted to hospital (93.6%) prior to their death. The median time between symptom onset and death was 8 days (interquartile range: 6‐11 days).

Vaccination information was available for 93.9% of deaths, and none were vaccinated for seasonal influenza. An underlying medical condition was recorded for 63.5% (n = 217) of all the influenza related deaths and for 62.2% of those aged 30‐64 years. The most commonly reported risk factor was cardiovascular disease (n = 55/342, 16.1%) followed by obesity (n = 37/342, 10.8%). Pregnancy was recorded in 2 cases (0.58%).

#### 3.1.5 Vaccine coverage

In 2015/16, an estimated 0.3% of the overall Ukraine population was vaccinated with the season's influenza vaccine. Vaccination was carried out mainly by local governments and varied by region from 0.01% in Zhytomyr up to 1.2% in the Dnipropetrovsk area.

### 3.2 Hazard assessment

#### 3.2.1 Virus characteristics

As of 8 February 2016, the WHO collaborating centres of the WHO Global Influenza Surveillance and Response System (GISRS) had characterised influenza A(H1N1)pdm09 viruses collected from over 30 countries globally since September 2015 and demonstrated that despite the genetic evolution of the A(H1N1)pdm09 virus, the majority of viruses remain antigenically closely related to the vaccine virus. This included 62 viruses submitted by both the UCDC and the L.V. Gromashevsky Institute.

Since 2009, the haemagglutinin (HA) genes of influenza A(H1N1)pdm09 have evolved into 8 genetic groups, with A/California/7/2009 representing group 1, and viruses in group 6 have formed clusters designated groups 6A, 6B and 6C. Phylogenetic analysis of the HA demonstrated that the HA genes of all the 62 analysed Ukrainian viruses belong to the 6B subgroup and cluster in the emerging subgroup 6B.1 with a smaller proportion of viruses belonging to other subgroup 6B.2, similar to the recent viruses from other parts of the world. The preliminary analysis for internal genes of the Ukraine viruses conducted by WHO collaborating centres does not reveal any amino acid substitutions currently known to be associated with pathogenicity or virulence.

All 62 A(H1N1)pdm09 viruses from Ukraine analysed were antigenically similar to the vaccine strain A/California/7/2009. All strains tested retained susceptibility to neuraminidase inhibitors oseltamivir and zanamivir. No evidence of reduced inhibition by molecular or phenotypic mechanisms to neuraminidase inhibitors was found.

## 4 DISCUSSION

The risk assessment of the whole WHO European Region concluded that there was higher influenza activity in eastern Europe in 2015/16 compared to western Europe and the European season was predominated by influenza A(H1N1)pdm09.^1^, ^2^ This country‐specific risk assessment found the 2015/16 influenza season in Ukraine (week 2015/40 until 2016/7) to be more severe than previous seasons. It was initiated by a steep increase in severe cases reported from late 2015. The season began earlier than usual but was consistent with the start of the season for the European Region.^1^, ^2^ The predominant strain circulating in Ukraine for 2015/16 was influenza virus A(H1N1)pdm09, which was associated with severe disease and deaths, particularly in younger adults.

These findings are consistent with the 2010/11 season, which was also dominated by early increases of influenza virus A(H1N1)pdm09 in western Europe and severe cases particularly among younger adults.^13^, ^14^ Influenza virus A(H1N1)pdm09 was found to be associated with more severe disease during the 2009 influenza (H1N1) pandemic, particularly affecting those with chronic health conditions. In Ukraine, there was no change in the proportion of severe cases with underlying medical conditions compared to previous seasons. However at 18%, this was much lower than findings of the US Influenza Hospitalization Surveillance Network for this season, where the majority of adult hospitalised cases had at least one reported underlying medical condition,^15^ and lower than findings from pooled analysis of hospitalised cases from different countries during the 2009 influenza (H1N1) pandemic (31.1%).^16^ Obesity was the most commonly reported underlying medical condition in Ukraine for laboratory‐confirmed SARI cases, which was a risk factor identified for severe outcome during the 2009 pandemic^16^ and has been documented further to be associated with higher risks of intensive care unit admission.^17^

There was a large increase in the number of laboratory‐confirmed influenza deaths reported for the early 2015/16 season, especially in the younger adult age group. The most commonly reported underlying conditions in those cases that died were cardiovascular disease and obesity, both previously described as independent risk factors associated with fatal outcomes for laboratory‐positive SARI in eastern Europe.^18^, ^19^ However, accurate estimates of deaths due to influenza are difficult to obtain.^20^ Deaths may occur due to complications occurring weeks after infection, or outside the healthcare system. It may therefore be possible to estimate death rates more accurately when vital statistics data become available. In week 7/2016, the European monitoring of excess mortality for public health action project (EuroMOMO) reported a pattern of excess all‐cause mortality among the age group of 15‐64 years from the end of 2015; this was slightly lower than 2014/15 but higher than 2013/14.^21^ However, this system mainly represents countries from western, northern and southern Europe and does not include Ukraine.

Although an east‐to‐west pattern of transmission was noted across the European Region this season,^1^, ^2^ there did not appear to be any clear pattern in the direction of transmission across Ukraine. The Russian Federation (east of Ukraine) also experienced a more severe season; both SARI and ARI increased slightly later than Ukraine at week 2/2016 and peaked later at week 5 or 6/2016. In Poland (north‐west of Ukraine), influenza activity was similar to the previous season and started to increase in week 1/2016. In Ukraine, influenza activity was reported across the country, with the capital Kyiv being the most affected area; this was consistent with geographical spread in 2014/15.

The severity of this season is likely to have been due to a combination of factors, in particular the dominance of influenza A (H1N1)pdm09, which was responsible for severe outcomes in particular in middle‐aged adults and the low vaccination coverage. Due to influenza A (H1N1)pdm09 predominantly affecting younger children in 2009, it created a smaller susceptible pool of children in subsequent seasons but a large pool of susceptible adult population remained.^14^ With a known increase in risk of complications from influenza infection with age,^22^ this therefore results in more severe cases. It has also been documented that a waning of pre‐existing immunity obtained against influenza A (H1N1)pdm09 since 2009 could occur within 6 months, thus leaving a further susceptible population.^23^ The seasonal influenza vaccination remains the best available protection against infection and is known to reduce secondary complications.^24^ The current seasonal vaccine was closely related to the predominant circulating virus and interim estimates of influenza vaccine effectiveness in Europe published by the influenza monitoring vaccine effectiveness (I‐MOVE) project showed moderate vaccine effectiveness of 46.3% (95% CI: 4.9%‐69.7%).^25^ A similar mid‐season vaccine effectiveness against influenza A(H1N1)pdm09 was found in the UK for 2015/16 (49.1%, 95% CI: 9.3‐71.5).^26^ However, vaccination coverage in Ukraine was very low and this may have resulted in a higher risk of complications and severe cases following influenza infection. None of the deceased cases reported were vaccinated. Vaccination coverage has been continually low in Ukraine. The seasonal vaccine is not mandatory but is recommended to certain risk groups such as those with chronic diseases, pregnant women, healthcare workers and persons over 60 years of age. Recommended vaccinations are mostly personally funded although some local governments do allocate money for vaccination of vulnerable groups and some large organisations vaccinate employees.

The early and steep increase in severe cases and deaths was associated with intensive media attention of this influenza season in Ukraine. This may have led to increased awareness of influenza infection among clinicians and the public. It is also possible that other respiratory pathogens such as RSV may have had a role in the increase in cases. However, the positivity rates of SARI cases sampled showed a steady increase peaking at 88%, confirming the role of influenza among SARI cases.

We did not conduct a full context assessment as part of this risk assessment and it was difficult to assess the role of the current conflict in eastern Ukraine on the severity of the influenza season. The surveillance systems used in this risk assessment did not cover the non‐government‐controlled areas to evaluate the influenza season impact. As a response to the current crisis, WHO has set up with collaborative partners, mobile emergency primary healthcare units (MEPUs) to provide healthcare services especially for the population close to the contact line. An Epiewarn reporting system was implemented by WHO, which included influenza‐like illness (ILI) and SARI reporting on a weekly basis for the 2015/16 season from week 5/2016 onwards. Influenza activity in this system was detected and peaked with about 5% of the MEPU patients reporting ILI, which then steadily declined.

As a preventive measure, the Ministry of Health in Ukraine recommended the closure of schools, nurseries and universities when the epidemic threshold was reached in mid‐January. Whether these measures have reduced the impact or the severity of the season remains to be evaluated.

The influenza season appeared to peak by week 04/2016 with cases subsequently started to decline, reducing the impact of the remaining influenza season. Analysis of the whole 2015/2016 season (week 40 to week 20) by the Ministry of Health Ukraine supported the findings in this risk assessment that the season was more severe in Ukraine with more cases of influenza and severe cases recorded than 2014/15.^27^ The high influenza activity in Ukraine was also noted in the WHO review of global influenza activity 2015/16.^28^ Influenza A(H1N1)pdm09 continued to be the predominant circulating strain for 2015/16 in Ukraine.

### 4.1 Limitations

Characteristics of influenza‐positive SARI cases, and deaths were collected using a tick box form with only positives recorded. When analysing these data, the assumption was made that if the condition was not indicated, then it was not present, and these results should therefore be interpreted with care. Further analysis is required to ensure that case definitions and sampling strategies were correctly applied throughout this epidemic; however, to our knowledge, there was no change in the surveillance systems between 2014/15 and 2015/16. We were unable to ascertain the data completeness of risk factors by clinics to do a full risk factor analysis or further statistical analysis. The small numbers of deaths in the 2014/15 season precluded statistical comparisons of characteristics and risk factors for mortality. We also acknowledge there are different risk assessment methodologies and this approach did not provide an overall quantification of the level of risk.

## 5 CONCLUSION AND RECOMMENDATIONS

The Ukraine 2015/16 influenza season was more severe than previous seasons. It was characterised by an earlier steep increase in severe cases and deaths than in the previous year, particularly in the younger adult population. The predominant circulating strain was A(H1N1)pdm09, which was closely related to the seasonal vaccine strains. There was no evidence of resistance to influenza antiviral drugs. However, vaccination coverage in Ukraine was low, which may have contributed to the higher number of severe cases.

Recommendations from this risk assessment were made and communicated at the time to support appropriate control measures. We recommended that awareness should be strengthened among healthcare personnel to suspect influenza in risk groups (pregnant women, individuals >6 months with certain chronic diseases, elderly persons, residents of institutions for older persons and the disabled, and children aged 6‐59 months). Severe disease may occur more frequently in adults due to a high proportion of circulating influenza A(H1N1)pdm09. Clinicians, especially those in emergency and intensive care units, should be enabled to provide appropriate critical care of patients with severe respiratory disease according to WHO recommendations. Administration of neuraminidase inhibitors within 48 hours of influenza symptom onset is recommended for persons at increased risk and exhibiting progressive disease, without waiting for diagnostic confirmation and irrespective of vaccination status. Surveillance information and viruses should continue to be shared through the European systems for timely risk assessment. To better respond during future influenza seasons, an end‐of‐season evaluation of current influenza surveillance systems within Ukraine should be considered to highlight strengths and areas for improvement. We recommend that healthcare workers and people who are most at risk of developing serious complications from influenza infection be vaccinated annually. Ukraine should attempt to increase seasonal vaccination coverage for the 2016/17 season through national vaccination campaigns.

## CONFLICTS OF INTEREST

None to declare.

## APPENDIX 1

### WHO REGIONAL OFFICE FOR EUROPE <2011 SARI CASE DEFINITION

Onset of the following symptoms ≤7 days prior to hospital admission: fever >38°C AND cough OR sore throat AND shortness of breath or difficulty in breathing.

For children aged <5 years, the WHO case definition for pneumonia and severe pneumonia is applied. Pneumonia: cough OR difficulty in breathing AND breathing faster than 40 breaths/min (12‐59 months); breathing faster than 50 breaths/min (2‐11 months); severe pneumonia: cough OR difficulty in breathing AND any of the following general severe signs: unable to drink or breastfeed OR vomits everything OR convulsions OR lethargy or unconsciousness OR chest indrawing or stridor in a calm child.

### WHO ARI CASE DEFINITION

Sudden onset of symptoms, and at least one of the following 4 respiratory symptoms: cough, sore throat, shortness of breath, coryza and a clinician's judgement that the illness is due to an infection.
